# Humidity and Deposition Solution Play a Critical Role in Virus Inactivation by Heat Treatment of N95 Respirators

**DOI:** 10.1128/mSphere.00588-20

**Published:** 2020-10-21

**Authors:** Nicole Rockey, Peter J. Arts, Lucinda Li, Katherine R. Harrison, Kathryn Langenfeld, William J. Fitzsimmons, Adam S. Lauring, Nancy G. Love, Keith S. Kaye, Lutgarde Raskin, William W. Roberts, Bridget Hegarty, Krista R. Wigginton

**Affiliations:** a Department of Civil & Environmental Engineering, University of Michigan, Ann Arbor, Michigan, USA; b Division of Infectious Diseases, Department of Internal Medicine, University of Michigan Health System, Ann Arbor, Michigan, USA; c Department of Urology, University of Michigan Health System, Ann Arbor, Michigan, USA; d Department of Biomedical Engineering, University of Michigan, Ann Arbor, Michigan, USA; Mount Sinai School of Medicine

**Keywords:** N95, bacteriophages, coronavirus, decontamination, droplet, fomite, heat, humidity, inactivation, influenza, mouse hepatitis virus, respirator

## Abstract

Supply shortages of N95 respirators during the coronavirus disease 2019 (COVID-19) pandemic have motivated institutions to develop feasible and effective N95 respirator reuse strategies. In particular, heat decontamination is a treatment method that scales well and can be implemented in settings with variable or limited resources. Prior studies using multiple inactivation methods, however, have often focused on a single virus under narrowly defined conditions, making it difficult to develop guiding principles for inactivating emerging or difficult-to-culture viruses. We systematically explored how temperature, humidity, and virus deposition solutions impact the inactivation of viruses deposited and dried on N95 respirator coupons. We exposed four virus surrogates across a range of structures and phylogenies, including two bacteriophages (MS2 and phi6), a mouse coronavirus (murine hepatitis virus [MHV]), and a recombinant human influenza A virus subtype H3N2 (IAV), to heat treatment for 30 min in multiple deposition solutions across several temperatures and relative humidities (RHs). We observed that elevated RH was essential for effective heat inactivation of all four viruses tested. For heat treatments between 72°C and 82°C, RHs greater than 50% resulted in a >6-log_10_ inactivation of bacteriophages, and RHs greater than 25% resulted in a >3.5-log_10_ inactivation of MHV and IAV. Furthermore, deposition of viruses in host cell culture media greatly enhanced virus inactivation by heat and humidity compared to other deposition solutions, such as phosphate-buffered saline, phosphate-buffered saline with bovine serum albumin, and human saliva. Past and future heat treatment methods must therefore explicitly account for deposition solutions as a factor that will strongly influence observed virus inactivation rates. Overall, our data set can inform the design and validation of effective heat-based decontamination strategies for N95 respirators and other porous surfaces, especially for emerging viruses that may be of immediate and future public health concern.

**IMPORTANCE** Shortages of personal protective equipment, including N95 respirators, during the coronavirus (CoV) disease 2019 (COVID-19) pandemic have highlighted the need to develop effective decontamination strategies for their reuse. This is particularly important in health care settings for reducing exposure to respiratory viruses, like severe acute respiratory syndrome coronavirus 2 (SARS-CoV-2), the virus that causes COVID-19. Although several treatment methods are available, a widely accessible strategy will be necessary to combat shortages on a global scale. We demonstrate that the combination of heat and humidity inactivates a range of RNA viruses, including both viral pathogens and common viral pathogen surrogates, after deposition on N95 respirators and achieves the necessary virus inactivation detailed by the U.S. Food and Drug Administration guidelines to validate N95 respirator decontamination technologies. We further demonstrate that depositing viruses onto surfaces when suspended in culture media can greatly enhance observed inactivation, adding caution to how heat and humidity treatment methods are validated.

## Abstract (precis)

Shortages of personal protective equipment, including N95 respirators, during the coronavirus (CoV) disease 2019 (COVID-19) pandemic have highlighted the need to develop effective decontamination strategies for their reuse. This is particularly important in health care settings for reducing exposure to respiratory viruses, like severe acute respiratory syndrome coronavirus 2 (SARS-CoV-2), the virus that causes COVID-19. Although several treatment methods are available, a widely accessible strategy will be necessary to combat shortages on a global scale. We demonstrate that the combination of heat and humidity inactivates a range of RNA viruses, including both viral pathogens and common viral pathogen surrogates, after deposition on N95 respirators and achieves the necessary virus inactivation detailed by the U.S. Food and Drug Administration guidelines to validate N95 respirator decontamination technologies. We further demonstrate that depositing viruses onto surfaces when suspended in culture media can greatly enhance observed inactivation, adding caution to how heat and humidity treatment methods are validated.

## INTRODUCTION

Effective decontamination of medical equipment is critical for controlling infectious diseases in clinical settings. This is heightened during pandemics, when shortages of personal protective equipment (PPE), such as N95 respirators, lead to occupational risks for health care workers. During the coronavirus (CoV) disease 2019 (COVID-19) pandemic, high demand for N95 respirators has led to interest in decontamination methods that do not compromise the effectiveness of the respirator. The application of heat, UV irradiation, and vaporized hydrogen peroxide are common decontamination treatments in medical settings, and research suggests that N95 respirators treated with these methods maintain their filtration integrity and fit (1–4). The U.S. Food and Drug Administration (USFDA) has issued an enforcement policy for face masks and respirators that presents specific recommendations for validation of PPE decontamination during the COVID-19 crisis. This policy calls for >3-log_10_ inactivation, validated using multiple viral pathogens, preferably coronaviruses (e.g., severe acute respiratory syndrome CoV [SARS-CoV], Middle East respiratory syndrome CoV [MERS-CoV], murine hepatitis virus [MHV], and transmissible gastroenteritis virus [TGEV]), and 6-log_10_ inactivation of mycobacteria or bacterial spores (5).

Arguably the simplest and most accessible approach to N95 respirator decontamination is to harness the biocidal activity of heat and moisture. Treating medical equipment with pressurized saturated steam in autoclaves for 1 h, for example, leads to high levels of virus inactivation (6–9); however, the high temperatures and pressures in autoclave sterilizers affect N95 respirator integrity (10, 11). In contrast, moist heat treatments at lower temperatures, from 60 to 90°C for 30 min or longer, do not affect filter performance and fit (12–15). To date, studies assessing inactivation of viruses on N95 respirators at elevated temperatures below 100°C have included limited viruses and conditions. Influenza viruses heated to 65°C with 85% relative humidity (RH) for 20 to 30 min resulted in >3-log_10_ inactivation (15, 16). Dry heat at 70°C for 1 h led to a >3-log_10_ inactivation of SARS-CoV-2 (2). In cases where heat treatment is not a feasible decontamination approach, the CDC recommends limited reuse of N95 respirators after incubation in paper bags at room temperature for an excess of 5 days (17). The justification for this recommendation is based on experiments evaluating the stability of SARS-CoV-2 on surfaces (18); however, the efficacy of this practice on N95 respirators has yet to be validated.

Previous virus inactivation studies that focus on other types of surfaces can inform N95 respirator decontamination strategies. Such studies suggest that both temperature and humidity can affect virus inactivation in droplets dried on porous or nonporous surfaces (19). Most surface inactivation studies have focused on virus inactivation under a limited range of environmentally relevant conditions (4 to 40°C) (20–22) and have reported greater virus inactivation at higher temperatures for MS2 bacteriophage (19, 23), enteric viruses (19, 23), coronaviruses (22, 24), and influenza A virus (25). Trends in humidity are not as easily discerned. While some surface inactivation studies have shown increased inactivation at elevated RHs (19, 22, 24, 25), others have reported that certain nonenveloped viruses are more stable at higher RHs (25). Limited work at elevated temperatures (55 to 65°C) has shown increased influenza virus inactivation as temperature and RH increase (26). A more systematic understanding of how humidity impacts inactivation for a range of viruses would aid the design of effective heat-based decontamination methods.

Cell culture medium suspensions are commonly used to generate droplets and aerosols to understand heat inactivation of viruses deposited on surfaces (18, 27, 28). Studies on virus inactivation at room temperature, however, have found that laboratory-made solutions (e.g., artificial saliva, cell culture media) are not representative of respiratory droplets and aerosols (25, 29–31). Indeed, deposition solution composition does appear to impact virus inactivation when virus particles are dried on surfaces, are present in droplets, or are present in aerosols (25, 30–34). Benbough (32) and Yang et al. (30), for example, observed that higher protein and salt concentrations positively influenced the persistence of viruses in aerosols and droplets under certain temperature and humidity conditions (30, 32). Protein content also had a protective effect for influenza viruses at median RHs (32). These studies have focused primarily on ambient conditions, and the potential impacts of deposition solution on virus inactivation at elevated temperatures has not yet been explored.

An improved understanding of how humidity, temperature, and deposition solution impact virus inactivation in dried droplets is critical to inform PPE decontamination and reuse practices in hospitals and other relevant settings. To address this, we studied virus decontamination on N95 respirator coupons using elevated heat and variable RHs for several RNA viruses deposited in droplets from four different solutions. We studied two diverse bacteriophages, MS2 and phi6, a mouse coronavirus (i.e., MHV), and a subtype H3N2 influenza virus (i.e., influenza A virus [IAV]) (Table 1). We focused on four RNA viruses because of their relevance to the current COVID-19 pandemic and because of their relevance for other respiratory viruses in clinical settings. Bacteriophage MS2, MHV, and IAV are single-stranded RNA viruses like SARS-CoV-2, whereas phi6 is a double-stranded RNA virus. As with SARS-CoV-2, phi6, MHV, and IAV are enveloped viruses. We included the two bacteriophages for several reasons, most importantly because their high stock concentrations facilitate experiments with large dynamic ranges. Additionally, the bacteriophages are biosafety level 1 organisms, quickly enumerated, and used extensively in surface decontamination studies, thus allowing for cross-study comparisons. We included an influenza virus because it is an important human respiratory virus that has been studied through N95 respirator decontamination processes (15, 16, 35, 36). The mouse coronavirus MHV is in the same genus as SARS-CoV-2 and is thus expected to exhibit a fate outside its host similar to that of SARS-CoV-2; it is important to note that MHV is not a sole respiratory virus and can infect the liver, gastrointestinal tract, and central nervous system (37, 38).

**TABLE 1:** Characteristics of SARS-CoV-2 and the viruses used in this study

Virus	Genome type^*a*^	Genome size	Particle size (nm)	Enveloped or nonenveloped
SARS-CoV-2	(+) ssRNA	29.9 kb	∼100	Enveloped
MS2	(+) ssRNA	3.6 kb	∼25	Nonenveloped
phi6	dsRNA	13.5 kbp	∼85	Enveloped
IAV	(–) ssRNA	13.6 kb	∼100	Enveloped
MHV	(+) ssRNA	31.3 kb	∼120	Enveloped

a (+), positive-sense; (–), negative-sense; ssRNA, single-stranded RNA; dsRNA, double-stranded RNA.

Our findings identify key parameters that drive virus inactivation on N95 respirator surfaces with heat and highlight the importance of deposition solution characteristics when validating decontamination methods. In particular, our results suggest that the common practice of depositing viruses using culture media may lead to a significant overestimation of the effectiveness of heat treatment for virus inactivation.

## RESULTS

### Virus inactivation improves with increasing RH and temperature.

**FIG 1:**
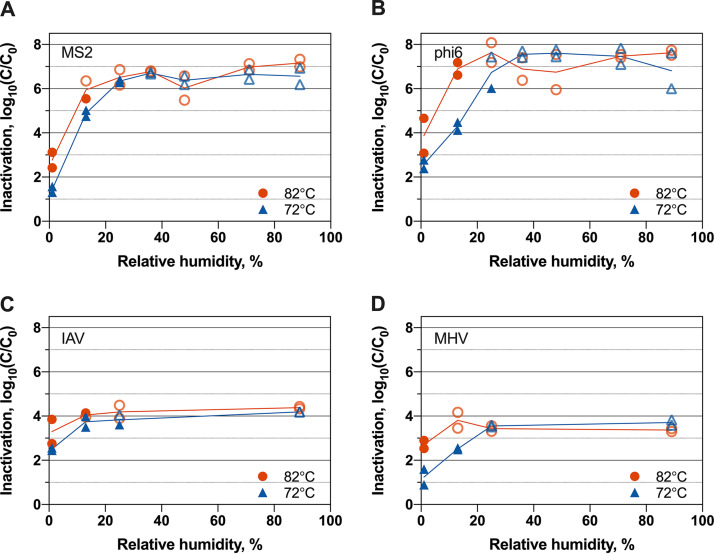
Inactivation of MS2 bacteriophage (A), phi6 bacteriophage (B), IAV (C), and MHV (D) at 72°C and 82°C for various RHs when viruses were suspended in culture media. Open faded symbols indicate virus inactivation beyond assay detection limits. Results from independent experimental replicates (*n* = 2) are shown for each virus under each condition.

To understand how temperatures and RHs impact inactivation through heat treatment, we deposited the four RNA viruses in culture media on N95 respirator coupons and treated the coupons for 30 min at 72°C and 82°C. An overall trend of increased virus inactivation was observed as temperature and RH increased (see Table S1 in the supplemental material). For all four viruses, inactivation was lowest at 1% RH at both 72°C and 82°C (Fig. 1). For treatments with RHs above 25% for 72°C and above 13% for 82°C, inactivation levels were beyond the dynamic ranges of our study for all viruses. Specifically, we observed >3.7 to > 8.1-log_10_ inactivation, depending on the virus. Consequently, we were unable to observe the inactivation trends at elevated RHs for any of the viruses tested. We note that the dynamic ranges were between 5.5 log_10_ and 8.1 log_10_ for the bacteriophages, ∼4.0 log_10_ for IAV, and ∼3.7 log_10_ for MHV. These dynamic ranges were determined as the log_10_ difference in titers between the no-treatment controls and the limits of detection of the enumeration assays (10 PFU/ml, 10 PFU/ml, 5 PFU/ml, and 20 50% tissue culture infective doses [TCID_50_]/ml for MS2, phi6, MHV, and IAV, respectively).

10.1128/mSphere.00588-20.4 TABLE S1 Log_10_ inactivation of all viruses across various heat and humidity treatments and deposition solutions. Download Table S1, PDF file, 0.05 MB. Copyright © 2020 Rockey et al. 2020 Rockey et al. This content is distributed under the terms of the Creative Commons Attribution 4.0 International license.

An increase in treatment temperature from 72°C to 82°C resulted in 1.3-log_10_ and 2.0-log_10_ average increases in inactivation for MS2 and phi6, respectively, across different RHs (Fig. 1). This trend of greater inactivation at 82°C than at 72°C was consistent across nearly all RH conditions for both viruses, although these increases were not always statistically significant (Table S2). Likewise, for IAV and MHV at 1% RH, the average inactivation increase was 1.1 log_10_ as temperature increased from 72°C to 82°C (Fig. 1). The influence of temperature on inactivation was not calculated for IAV and MHV above 1% RH, as assay detection limits were exceeded.

10.1128/mSphere.00588-20.5 TABLE S2 Results of unpaired *t* tests comparing virus log_10_ inactivation values at 72°C and 82°C. Download Table S2, PDF file, 0.04 MB. Copyright © 2020 Rockey et al. 2020 Rockey et al. This content is distributed under the terms of the Creative Commons Attribution 4.0 International license.

RH strongly affected virus inactivation. For a given temperature, all four viruses demonstrated increased inactivation with increasing RH for all experiments that fell within the virus assay dynamic ranges (Fig. 1). Among the bacteriophages, a 10% increase in RH corresponded to 2.5-log_10_ and 2.4-log_10_ average increases in inactivation for MS2 and phi6, respectively, across all temperatures within assay limits (Table S3). MS2, for example, underwent an average of 1.4-log_10_ inactivation at 1% RH and an average of 4.9-log_10_ inactivation at 13%. MHV and IAV inactivation increased by 1.2 log_10_ and 1.3 log_10_, respectively, from 1% to 13% RH at 72°C.

10.1128/mSphere.00588-20.6 TABLE S3 Results of unpaired *t* tests comparing virus inactivation values at different RHs. Download Table S3, PDF file, 0.05 MB. Copyright © 2020 Rockey et al. 2020 Rockey et al. This content is distributed under the terms of the Creative Commons Attribution 4.0 International license.

No consistent trend in inactivation was observed between mammalian viruses and the bacteriophages. For example, the four viruses demonstrated similar inactivation levels at 1% RH at both 72°C and 82°C, but IAV and MHV were inactivated less than the bacteriophages at 72°C and 13% RH (Tables S1 and S4). The small dynamic range of IAV and MHV limited our ability to observe inactivation trends for these viruses over a broad range of temperature and humidity conditions.

10.1128/mSphere.00588-20.7 TABLE S4 Results of unpaired *t* tests comparing levels of inactivation of different viruses. Download Table S4, PDF file, 0.05 MB. Copyright © 2020 Rockey et al. 2020 Rockey et al. This content is distributed under the terms of the Creative Commons Attribution 4.0 International license.

The two cell culture medium formulations, termed DMEM-A and DMEM-B, used in the above-described experiments consisted of Dulbecco’s modified Eagle’s medium (DMEM) and contained different supplementary ingredients (Table S5). Control experiments with phi6 and MS2 deposited in both of these medium types and treated at 72°C and 13% RH demonstrated that similar levels of inactivation were experienced in either DMEM composition (Fig. S1).

10.1128/mSphere.00588-20.1 FIG S1 Log_10_ inactivation of bacteriophages at 72°C and 13% in DMEM-A and DMEM-B. Results from independent experimental replicates (*n* = 2) are shown for each virus under each condition. Download FIG S1, TIF file, 0.2 MB. Copyright © 2020 Rockey et al. 2020 Rockey et al. This content is distributed under the terms of the Creative Commons Attribution 4.0 International license.

10.1128/mSphere.00588-20.8 TABLE S5 Compositions of DMEM-A and DMEM-B used for deposition solutions. Download Table S5, PDF file, 0.07 MB. Copyright © 2020 Rockey et al. 2020 Rockey et al. This content is distributed under the terms of the Creative Commons Attribution 4.0 International license.

### Deposition solution influences virus inactivation.

**FIG 2:**
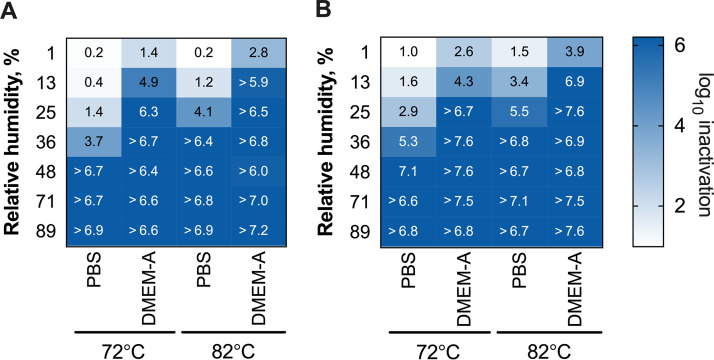
Inactivation of bacteriophages MS2 (A) and phi6 (B) at various high temperatures and RHs with PBS and DMEM-A deposition solutions. Presented values are the average log_10_ inactivation of independent experimental replicates (*n* = 2) for each condition. Individual replicate data are provided in Table S1 in the supplemental material for MS2 and phi6, respectively.

To assess potential effects of using tissue culture medium as the deposition solution on observed virus inactivation, we conducted phi6 and MS2 experiments over the same range of RHs and temperatures in a phosphate-buffered saline (PBS) deposition solution. We did not include MHV and IAV in these experiments due to the challenges of resuspending MHV and IAV in PBS without decreasing stock concentrations and thus decreasing the experiment dynamic ranges. For both phages, the deposition solution had a profound effect on inactivation (Fig. 2). Bacteriophages deposited in DMEM-A were inactivated significantly more than when they were deposited in PBS under all conditions for MS2 and under three out of four conditions for phi6 (Table S6). The most striking difference occurred at 25% RH for 72°C treatments. At 72°C and 25% RH, for example, only 1.4-log_10_ and 2.9-log_10_ inactivations were observed for MS2 and phi6, respectively, when deposited in PBS. When the bacteriophages were deposited in DMEM-A and treated under the same conditions, the observed inactivations were 6.4 log_10_ and >6.7 log_10_. This is approximately a 4- to 5-log_10_ difference in inactivation resulting solely from the type of deposition solution. With either deposition solution, phi6 was more susceptible to heat treatment than MS2. When deposited in PBS solution, phi6 was inactivated, on average, 1.4 log_10_ more than MS2, although significance could not be confirmed for all temperature and RH conditions (Table S4). The difference in inactivation between MS2 and phi6 was less pronounced when viruses were deposited in DMEM-A. There, phi6 was inactivated, on average, 0.6 log_10_ more than MS2. We note that differences between phi6 and MS2 inactivation were not due to differences in their respective stock solutions, because the two viruses were deposited in a single mixed stock solution.

10.1128/mSphere.00588-20.9 TABLE S6 Results of unpaired *t* tests comparing levels of virus inactivation across different deposition solutions. Download Table S6, PDF file, 0.05 MB. Copyright © 2020 Rockey et al. 2020 Rockey et al. This content is distributed under the terms of the Creative Commons Attribution 4.0 International license.

10.1128/mSphere.00588-20.10 TABLE S7 Deposition solutions used in experiments. Download Table S7, PDF file, 0.04 MB. Copyright © 2020 Rockey et al. 2020 Rockey et al. This content is distributed under the terms of the Creative Commons Attribution 4.0 International license.

**FIG 3:**
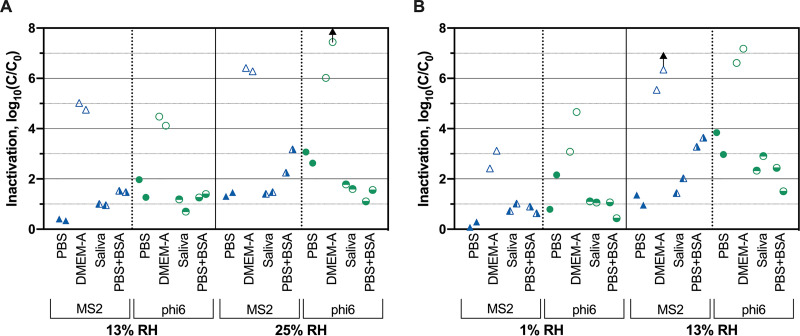
Susceptibility of MS2 and phi6 to heat and RH treatment at 72°C (A) and 82°C (B) when deposited in four matrices. Arrows indicate virus inactivation beyond detection limits. Results from independent experimental replicates (*n* = 2) are shown for each virus under each condition.

To determine if protein content in IAV contributed to the observed differences in virus inactivation between DMEM-A and PBS, we tested MS2 and phi6 inactivation with a PBS deposition solution that was supplemented with the same amount of BSA as was present in DMEM-A. Bacteriophage inactivation in PBS plus BSA deposition solution was significantly less than in DMEM-A for both MS2 and phi6 across nearly all conditions tested (Fig. 3; Table S6), with average reductions of 3.0 log_10_ and 3.7 log_10_ for MS2 and phi6, respectively. With respect to the specific effect of BSA in the deposition solution, MS2 was inactivated 1.3 log_10_ more when the PBS deposition solution contained BSA. Two out of four of the temperature/RH conditions exhibited statistically significant differences (Table S6). For phi6, the opposite trend was observed; adding BSA to the PBS deposition solution resulted in an average of 1.0 log_10_ less inactivation. Here, only one of four conditions tested resulted in a statistically significant difference (Table S6). Overall, these results showed that virus inactivation in PBS plus BSA was more similar to inactivation in PBS than in DMEM-A for phi6 and MS2.

To better represent virus-containing droplets present on N95 respirators, we tested MS2 and phi6 inactivation in freshly collected human saliva sterilized using UV treatment. Phage inactivation in saliva was within 0.7 log_10_ of inactivation in PBS, on average (Fig. 3). Inactivation of the two viruses deposited in DMEM-A was significantly greater than inactivation when deposited in saliva across nearly all treatments (3.6 log_10_ and 3.5 log_10_ greater for MS2 and phi6, respectively) (Table S6). On average, inactivation levels of MS2 deposited in saliva were 0.5 log_10_ larger than inactivation levels in PBS (Fig. 3). For phi6, inactivation in saliva was 0.8 log_10_ less than in PBS (Fig. 3). These results indicate that deposition in saliva is more similar to deposition in PBS than in DMEM-A.

### Virus inactivation under ambient conditions.

**FIG 4:**
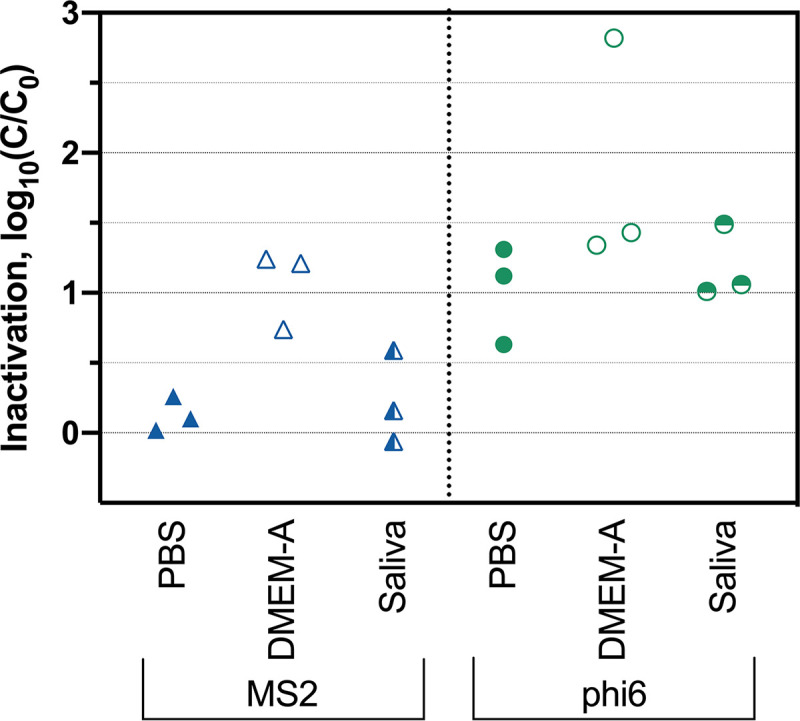
Inactivation of MS2 and phi6 after 24 h at 20°C and 36% RH. Results from independent experimental replicates (*n* = 3) are shown for each virus in each deposition solution.

To assess whether virus inactivation under ambient conditions was also affected by deposition solution, we tested bacteriophage inactivation at room temperature (20°C) and 36% RH using different deposition matrices. This RH and temperature were selected at the lower end of standard thermohygrometric ranges for health care facilities (39). After 24 h, we observed relatively low levels of inactivation (<2 log_10_ on average) for both MS2 and phi6 in all deposition matrices (Fig. 4). MS2 inactivation in DMEM-A was significantly higher than in either PBS (*P* = 0.0061) or saliva (*P* = 0.029). Although the average inactivation was higher for phi6 deposited in DMEM-A than for phi6 deposited in PBS and saliva, the differences were not statistically significant.

## DISCUSSION

Heat remains a widely accessible strategy for decontamination of PPE due to the ubiquity of ovens that can achieve sufficient temperatures and the ease of translating and communicating effective protocols. Furthermore, the temperatures and treatment times used in this study do not negatively impact respirator integrity (12–15), which is essential for this method’s applicability. To support the use of effective N95 decontamination approaches during the COVID-19 pandemic, the USFDA provided a number of emergency use authorizations (EUAs) and published recommendations for evaluating N95 decontamination methods. An early recommendation published by the USFDA for the decontamination and reuse of respirators for single users suggested demonstrating ≥3-log_10_ inactivation of viruses, specifically those related to SARS-CoV2 (e.g., SARS-CoV, MERS-CoV, and TGEV), along with ≥6-log_10_ inactivation of either mycobacterial or bacterial spores. Our results demonstrate that heat treatment (72°C and 82°C) with moderate-to-high RHs (above 25%) for 30 min can inactivate at least 3 log_10_ of MS2, phi6, IAV, and MHV. However, the USFDA guidance was replaced with recommendations to demonstrate ≥6-log_10_ inactivation of three nonenveloped viruses and ≥6-log_10_ inactivation of two Gram-positive bacteria and two Gram-negative bacteria (40). The recommended 6-log_10_ inactivation is easily achievable for MS2 and phi6 with the heat and high-RH treatments. The latest guidance does not address which viruses should be tested or which deposition solutions should be used. In addition to identifying the best conditions for heat and humidity decontamination of N95 respirators, our work sought to identify how different surrogate viruses and deposition solutions might affect results.

### Influence of elevated temperature and humidity on virus inactivation.

At first glance, our results suggest that increasing temperature improves virus inactivation. When temperature was increased from 72°C to 82°C at a constant RH, average inactivation increases of 1.2 log_10_, 1.6 log_10_, 0.8 log_10_, and 1.5 log_10_ were observed for MS2, phi6, IAV, and MHV, respectively. Other studies have observed improved virus inactivation at increasing temperatures, both at elevated temperatures (i.e., 55°C versus 65°C) (26) and at ambient temperatures ranging from 4 to 40°C (19, 22, 23). Interestingly, when our virus inactivation results are presented as a function of absolute humidity, the effect of increased temperature on inactivation is no longer evident (see Fig. S2 in the supplemental material). In other words, the improved inactivation observed for 82°C compared to that for 72°C was driven more by the added water content than by the temperature. In several studies that have taken a modeling approach, absolute humidity was deemed a better predictor of IAV inactivation than temperature and RH both at elevated temperatures (26) and in ambient temperature ranges (41, 42). Prussin et al., however, reported that the conclusions about absolute humidity versus RH can vary depending on the type of model used (43). Overall, a mechanistic understanding of what drives virus inactivation on surfaces and in aerosols at various temperatures and humidities remains elusive. Due to the fact that most studies on the effects of humidity on virus inactivation report their findings as a function of RH (19, 22, 23, 25, 27, 32, 44, 45), we chose to present humidities primarily as RH for this study.

10.1128/mSphere.00588-20.2 FIG S2 Log_10_ virus inactivation as a function of absolute humidity for MS2 bacteriophage (A), phi6 bacteriophage (B), IAV (C), and MHV (D). Open faded symbols indicate virus inactivation beyond assay detection limits. Results from independent experimental replicates (*n* = 2) are shown for each virus under each condition. Download FIG S2, TIF file, 1.1 MB. Copyright © 2020 Rockey et al. 2020 Rockey et al. This content is distributed under the terms of the Creative Commons Attribution 4.0 International license.

Virus inactivation improved as RH increased from 1% to 48% in our experiments at 72°C and 82°C (detection limits were exceeded under all conditions above 48%). A similar RH effect was observed previously for elevated heat treatment of IAV deposited on steel coupons (26). The impacts of RH on virus inactivation at lower temperatures are not consistent in the literature. In several studies where diverse viruses have been dried on surfaces and exposed to 4 to 40°C, increasing RH from between 20 and 30% to between 50 and 80% also increased inactivation (19, 22, 25). Other studies have observed the opposite trend when viruses were dried on surfaces (25) or were present in aerosols (44, 45), with less inactivation at high RH than at low RH. Phi6 and IAV inactivation at ambient temperatures in droplets exhibited yet a different trend, with greatest inactivation at RH levels ranging from 60 to 85% and decreasing at lower and higher RHs (30, 43, 46). The reasons for these discrepancies are not clear but may be in part due to differences in the deposition solutions and drying conditions. We suggest that future studies on the impact of temperature and humidity always include a virus that is simple to measure and widely accessible (e.g., bacteriophage MS2) in addition to their viruses of interest (e.g., SARS-CoV-2). Doing so would facilitate cross-study comparisons of various viruses and conditions.

### Virus inactivation under ambient conditions.

At room temperature and 40% RH for 24 h, <2-log_10_ inactivation of MS2 and phi6 bacteriophages on N95 respirators was observed, regardless of the deposition solution used. Other studies observed ≤1-log_10_ inactivation of MS2 and phi6 at ambient temperature and RH over 24 h (27, 47). We observed an additional 0.9-log_10_ inactivation of phi6 compared with that of MS2, although this difference was not statistically significant. Our tests did not assess the impact of time on ambient condition treatment; we therefore cannot predict the inactivation levels that would be reached when N95 respirators are left at ambient conditions for 5 days, as specified by CDC guidelines (17). However, previous studies have assessed the persistence of MS2 (27), phi6 (47), and various influenza strains (48) on surfaces over extended time periods. If the rates of inactivation from these studies hold true, 5.5-log_10_ and 4.8-log_10_ inactivation of phi6 and MS2, respectively, on N95 respirators and 13.6-log_10_ IAV inactivation on porous surfaces (48) can be expected after 5 days at ambient temperatures and 30 to 60% RH. Research observing surface stability of clinically relevant viruses at ambient conditions, including IAV and SARS-CoV-2, suggests that coronaviruses are not as persistent as IAV or the bacteriophages studied here (18, 35). More work is needed with multiple viruses, with longer storage times, and in saliva or other respiratory fluids to determine if room temperature storage is an effective decontamination strategy for N95 respirators.

### Virus-specific inactivation trends.

The results of this study demonstrate the importance of assessing inactivation for a diverse set of surrogate viruses when inactivation experiments cannot be performed with the virus of interest because it is not culturable or requires high biosafety containment. In our study, we focused most on the enveloped phi6 bacteriophage and nonenveloped MS2 bacteriophage. To test viruses more similar to SARS-CoV-2, we also included IAV and MHV in a subset of experiments; however, these viruses, like SARS-CoV-2, are difficult to propagate to high titers, thus limiting the experimental dynamic ranges and deposition solutions. Our broad data set for MS2 and phi6 under different heat/humidity conditions (Fig. 2) and in different matrices (Fig. 3) suggest that enveloped phi6 deposited on N95 respirators is more susceptible to heat and humidity than nonenveloped MS2 under nearly all conditions tested. Previous research on viruses dried on surfaces and exposed to room temperature also found increased persistence of nonenveloped viruses compared to enveloped viruses (49, 50). As viruses dry on surfaces, it has been suggested that the air-water interface may damage the lipid membranes of enveloped viruses (19, 30), and the increased salt concentration can cause the lipid membrane to become rigid (51). Moreover, some studies observed that increased salt concentrations can protect nonenveloped viruses (30, 32).

We did not observe that enveloped mammalian IAV and MHV were consistently more susceptible to inactivation than nonenveloped MS2 (Fig. 1). In fact, MHV was less susceptible than both bacteriophages to heat treatment at 72C and 13% RH when deposited in culture media (Fig. 1). We note that our dynamic ranges for MHV and IAV were much smaller than those for MS2 and phi6. Furthermore, MHV and IAV experiments were conducted only when their culture media were used as deposition solutions. These limitations of the IAV and MHV experiments affect our ability to observe major trends in their relative susceptibilities. Nonetheless, the results from experiments with all four viruses suggest that bacteriophages are not always conservative surrogates for IAV and coronavirus inactivation through heat and humidity treatment. This brings into question the USFDA’s guidelines for using nonenveloped viruses as conservative surrogates for pathogenic viruses on N95 respirator decontamination. A review by Yang and Marr on virus survival in aerosols indicates that the presence of a lipid envelope is not solely responsible for virus susceptibility to inactivation; they suggest other virus characteristics, such as virus infection mechanisms and protein stability, are necessary to explain observed inactivation levels (52). To better account for these differences, a “cocktail” approach to assessing virus inactivation may be most suitable, using a wide range of surrogate viruses that have various characteristics in common with the viral pathogens of interest.

### Effects of deposition solution.

Our results demonstrate that the deposition solution used to apply viruses to N95 respirators greatly impacts virus inactivation at both elevated and ambient temperatures. At elevated temperatures, both MS2 and phi6 were inactivated much more when deposited in their culture media than when deposited in PBS (Fig. 2 and 3). At room temperature, only MS2 was inactivated to a statistically greater extent when deposited in its culture medium (Fig. 4). There has been limited prior work comparing levels of virus survival in PBS and culture media. Unlike with our results, Yang et al. observed that IAV deposited in culture media supplemented with fetal calf serum exhibited less inactivation than viruses deposited in PBS when exposed to room temperature and RHs from 20% to 60% (30). This discrepancy may be due to the fact that the deposition solution was allowed to dry before heat treatment in our study, whereas Yang et al. measured inactivation while viruses were still suspended in droplets. Consistent with this hypothesis, Sizun et al. observed that human coronaviruses OC43 and 229E in suspension exhibited less inactivation in PBS than in culture media with fetal bovine serum at room temperature (21).

There are multiple possibilities that may explain the observed differences in inactivation between PBS and culture medium deposition solutions. Studies with aerosols or droplets have suggested that increasing salt concentrations in media may increase inactivation (30, 32); however, the PBS and DMEM-A solutions in our study had similar salt contents (Tables S5 and S7). Proteins in the deposition solution may have a protective effect on viruses (29, 30). Our results, however, show that inactivation with viruses deposited in PBS plus BSA was significantly less than when applied in DMEM-A, which contains BSA at the same levels (Fig. 3). Therefore, protein content also does not appear to explain the observed differences. Another possible explanation is that the l-glutamine present in DMEM-A degrades into glutamate and ammonia (53, 54). Ammonia is known to cause virus inactivation in solution (55), although further work is needed to test this hypothesis in dried droplets and at elevated temperatures.

Ultimately, it is important to understand the extent of inactivation when viruses are deposited in the actual matrices found on N95 respirators. The limited studies that have compared artificial and more realistic deposition matrices (e.g., human saliva) suggest that laboratory-made solutions (e.g., cell culture media or artificial saliva) are not fully representative of respiratory droplets and aerosols (29, 34, 35, 56). For this reason, we tested human saliva as a more realistic deposition solution and compared the results to those obtained with the other deposition solutions. Our results show that MS2 and phi6 were significantly more susceptible to inactivation in DMEM-A than in saliva (Fig. 3). Furthermore, the PBS deposition solution provided the results most similar to those of the saliva deposition solution. The results using the two bacteriophages suggest that PBS may be an appropriate deposition solution for studying virus inactivation on surfaces. Additional experiments will need to be conducted with a broader set of viruses to determine if a PBS deposition solution is always representative of saliva.

In light of our results, it is important to consider the deposition solution when reexamining earlier reports of virus inactivation on N95 respirators and other materials. Heat inactivation studies and room temperature inactivation studies often either use culture media to generate droplets and aerosols or do not explicitly state which solutions are used (2, 15, 16, 18, 27, 28). Given that our observed MS2 and phi6 inactivation trends hold for other viruses, then inactivation in several studies using culture media for droplet deposition may overestimate inactivation relative to what occurs when viruses are dried on N95 respirators in saliva or respiratory fluids. A recent study of SARS-CoV-2 inactivation on N95 respirators, for example, reported 3-log_10_ virus inactivation at 70°C under “dry heat” conditions for 60 min (2). Although the deposition solution was not explicitly stated in this study, inactivation of SARS-CoV-2 may have been significantly overestimated if a culture medium was used for deposition. This is of critical importance for clinical settings, because these results may lead health care workers to disinfect respirators at 70°C for 60 min in an oven without controlled humidity, whereas our results at 72°C and 1% RH for 30 min suggest that little inactivation would take place under these conditions.

### Conclusions.

Our work demonstrates the virus inactivation efficacy of heat and humidity treatments for N95 respirator decontamination. The USFDA’s recommended 6-log_10_ inactivation of viruses was easily achievable for bacteriophages MS2 and phi6 with this heat-humidity paradigm. Likewise, although we were limited by the dynamic range of our assays, the more clinically relevant virus surrogates, MHV and IAV, resulted in at least 3-log_10_ inactivation under the same conditions. Low (<25%) RH treatments at the same temperatures were not as effective. We note that research to validate the inactivation of bacterial and fungal pathogens in addition to viral pathogens must be completed to entirely satisfy the USFDA’s guidelines for N95 respirator decontamination technologies for all pathogens. We also observed that inactivation was strongly influenced by the deposition solution. Dried virus droplets in cell culture media were inactivated significantly more than in any other deposition solution (PBS, PBS plus BSA, saliva). These findings suggest that virus inactivation may be vastly overestimated when using culture media as the deposition solution in surface disinfection studies. We suggest the use of deposition solutions more similar to human saliva or respiratory fluid in virus inactivation experiments to ensure representative results.

Hospitals and other health care settings can expect extensive virus inactivation of N95 respirators through heat treatment for at least 30 min at 72°C or 82°C and RH above 50%. High-humidity heat treatment is particularly appealing, as it can readily be adapted and scaled to a range of settings, from health care facilities to private residences. Further, implementation is equally suitable for health care systems or individuals without access to specialized equipment, including those in low- to middle-income countries. These results provide timely and useful information for efficacious N95 respirator decontamination, enabling reuse when necessary due to shortages.

## MATERIALS AND METHODS

### Virus stocks and enumeration.

Table 1 shows characteristics of the viruses used in this study along with the same characteristics of SARS-CoV-2. MS2 bacteriophage and its Escherichia coli host were obtained from the American Type Culture Collection (ATCC 15597). Bacteriophage phi6 and its Pseudomonas syringae pv. phaseolicola host were provided by Linsey Marr at Virginia Tech. MHV strain A59 and its murine delayed brain tumor (DBT) host cell line were provided by Julian Leibowitz at Texas A&M Health Science Center College of Medicine. For IAV, we used a recombinant virus that expresses the luciferase reporter in infected cells. This system allows for rapid titering based on light emission in infected cells. The virus is a 6+2 reassortant, in which the genomic segments encoding the surface hemagglutinin (HA) and neuraminidase (NA) are derived from A/Wisconsin/67/2005 (H3N2) and the remaining six segments are derived from A/WSN33 (H1N1). In this case, the segment 3 RNA encodes a polymerase acidic (PA) protein that is fused to the NanoLuc reporter (66). Recombinant viruses were harvested after transfection of HEK 293T/MDCK-SIAT1 cocultures with plasmids expressing the genomic RNA and proteins of all 8 segments. Rescued viruses were passed once on MDCK-SIAT1 cells at a multiplicity of infection (MOI) of 0.05 to obtain a passage 1 (P1) stock.

### Virus propagation and purification.

The MS2 viruses were propagated and their titers were determined based on established methods (57, 58). The MS2 lysate was concentrated with polyethylene glycol 8000 (Fisher Scientific, catalog [cat.] no. BP2331), treated with chloroform, and filter sterilized with a 0.22-μm polyethersulfone (PES) membrane filter (Celltreat Scientific, cat. no. 229746). Propagated phi6 was filtered with a 0.22-μm PES membrane filter, concentrated by tangential-flow filtration (Millipore, cat. no. C1975) with a 30-kDa cellulose filter (Millipore, cat. no. PXC030C50), purified by sucrose gradient ultracentrifugation, and filtered sterilized with 0.22-μm PES membranes (59). The MS2 and phi6 experimental stocks were combined, resulting in a single bacteriophage stock with each virus present at 10^11^ PFU/ml. The stocks were aliquoted and stored at −80°C prior to use.

For MHV propagation and enumeration, DBT cells were grown in DMEM (Lonza, cat. no. 12614F) supplemented with 10% horse serum (Life Technologies, cat. no. 26050088), 1% penicillin streptomycin (Invitrogen, cat. no. 15140122), and 1% l-glutamine (Invitrogen, cat. no. 25030081) at 37°C and 5% CO_2_. Cells were infected at an MOI of ∼0.01 when they were 75% confluent and then incubated with virus for 24 h. The cell suspension was frozen at −80°C, thawed, and centrifuged at 3,000 × *g* for 15 min at 4°C, and then the supernatant was recovered. The resulting virus stock (∼10^6^ PFU/ml) was filtered with a 0.22-μm PES membrane and stored in single-use aliquots at −80°C. For plaque assay enumeration, samples were diluted in DMEM-B (DMEM with 2% horse serum, 1% penicillin and streptomycin, and 1% l-glutamine) and applied to confluent DBT cells washed with 1× PBS (Invitrogen, cat. no. 10010023). After 1 h of incubation at room temperature with rocking, the virus suspension was removed from monolayers and an overlay of 1.6% agar mixed 1:1 with a 2× minimum essential medium (MEM; Quality Biological, cat. no. 115073101) containing 5% horse serum, 10 mM HEPES (Lonza, cat. no. 17737E), 1× MEM nonessential amino acids (Invitrogen, cat. no. 11140050), 2% l-glutamine, and 2% penicillin streptomycin was applied. Infected cells were then incubated for 48 h at 37°C and 5% CO_2_ before being stained with neutral red solution (Sigma-Aldrich, cat. no. N2889) diluted in 1× PBS to a 0.01% final concentration. Plaque assays were conducted in triplicate, and a negative medium control was performed with each assay.

IAV propagation was performed in DMEM-A (Gibco, cat. no. 11965092) with 25 mM HEPES, pH 7.2 to 7.5, 0.1875% fraction V bovine serum albumin (BSA; Gibco, cat. no. 15260037), 1% penicillin and streptomycin (10,000 U/ml; Gibco, cat. no. 15140122), and 2 μg/ml TPCK (tosylsulfonyl phenylalanyl chloromethyl ketone-treated trypsin [Worthington Biochemical Corporation, cat. no. LS003740]). IAV stocks were stored as single-use aliquots in 0.5% glycerol at −80°C. IAV cells were enumerated in MDCK-SIAT1 cells by endpoint dilution using IAV titer medium, which contained 1% BSA but otherwise had the same components as DMEM-A. Eighteen hours postinfection, media were aspirated and replaced with IAV titer medium containing 7.5 μM ViviRen live-cell substrate (Promega, cat. no. E6491). Light emission was measured using a BioTek Synergy HTX luminometer using the following settings: a 3-min dark adapting hold, emission hole, top optics position, gain of 160, integration time of 1.00 s, and read height of 2.24 mm. The room temperature A well was considered positive for infection if the relative light units (RLU) were greater than or equal to twice the average background RLU from eight mock-infected wells.

### Droplet deposition.

The combined bacteriophage stock was suspended to a final concentration of approximately 10^10^ PFU/ml in various deposition solutions. These deposition solutions (see Table S7 in the supplemental material), each used in a subset of experiments, included 1× PBS, DMEM-A, with the exception that no trypsin was added (Table S5), DMEM-B (Table S5), PBS with 0.1875% BSA (PBS plus BSA), or human saliva. For each saliva experiment, fresh saliva was collected from a volunteer and UV treated to sterilize (60, 61). Volunteers did not eat within 2 h prior to collection and rinsed their mouth with water 10 min before collection (62, 63). Saliva was collected in two wells of a 12-well plate in a thin layer. The saliva was immediately treated for 5 min using a custom-built collimated beam equipped with 0.16 mW cm^−2^ UV_254_ lamps (model G15T8; Philips). Lamp intensity was measured using chemical actinometry (64, 65). MHV (∼10^6^ PFU/ml) in DMEM-B (Table S5) and IAV (∼10^7^ TCID_50_/ml) in DMEM-A (Table S5) were used for droplet deposition. We used the highest virus concentrations possible for droplet deposition to maximize the log_10_ inactivation that we could observe through decontamination treatments.

We deposited 25 2-μl droplets across the 2.54-cm-diameter circular coupons, resulting in a total of 50 μl deposited in each experiment. The 2-μl droplet volumes are similar to volumes used in other droplet studies (21, 22, 28, 36). The coupons were generated from 3M 1860 N95 respirators with a 2.54-cm arch punch. The droplets were then allowed to dry in a biosafety cabinet at room temperature and ambient RH for approximately 1 h. Details of a control experiment to determine whether extended coupon dry times impacted virus inactivation are included in the supplemental material (Text S1). Each treated coupon had a corresponding control coupon that was prepared at the same time as the treated coupon but was maintained at ambient conditions during the experiments.

10.1128/mSphere.00588-20.3 TEXT S1 Descriptions of (i) a control experiment to test the potential impact of drying time on virus reduction and (ii) methods for assessing virus recovery from N95 respirator coupons. Download Text S1, PDF file, 0.06 MB. Copyright © 2020 Rockey et al. 2020 Rockey et al. This content is distributed under the terms of the Creative Commons Attribution 4.0 International license.

### Temperature and humidity controlled oven.

The temperature- and humidity-controlled oven (TestEquity 123H temperature/humidity chamber) used in all experiments was calibrated for temperature to be accurate within 2°C and for RH to be accurate within 5%. A second external instantaneous hygrometer probe (Fisher Scientific, cat. no. 116617B) rated to be accurate to within 0.2°C and 1.5% RH was also used to monitor the oven. The experiments were designed to test regular intervals of RH using the humidity chamber’s readout (i.e., 10%, 20%, 30%, 40%, 50%, 70%, 90%); however, due to the lower error associated with the external hygrometer, external hygrometer values (1%, 13%, 25%, 36%, 48%, 71%, 89%) were used in the data analysis.

### Heat- and RH-controlled experiments.

The heat- and humidity-controlled oven was set to the desired RH and temperature for at least 30 min prior to use. Dried coupons were immobilized on a coated metal test tube rack with metal binder clips. The metal rack with coupons was then transferred to the oven at the predetermined temperature and humidity settings. Treatment times were started when the oven RH was within 1% of the target RH for 13 to 71% RH and within 5% of target RH for 1% and 89% RH. The oven reached these conditions within 5 min. Heat decontamination experiments with phi6 and MS2 deposited in PBS and DMEM-A were conducted at 72°C and 82°C and 1%, 13%, 25%, 36%, 48%, 71%, and 89% RH. Experiments with IAV and MHV were carried out in duplicate for a subset of experimental conditions (i.e., temperatures of 72°C and 82°C, each at 1%, 13%, 25%, 48%, and 89% RH). Experiments with additional deposition solutions, including human saliva and PBS with BSA, were carried out for MS2 and phi6 at 72°C with 13% and 25% RH and at 82°C with 1% and 13% RH. These additional experiments were not conducted with MHV and IAV due to issues with resuspending them in saliva and PBS without further reducing the dynamic range of the experiments. DMEM-A and DMEM-B contained different supplementary ingredients; to assess the possible effects of these differences on observed virus inactivation, duplicate control experiments with MS2 and phi6 at 72°C and 13% RH were carried out in both DMEM-A and DMEM-B.

Twenty-four-hour experiments were conducted with MS2 and phi6 to assess whether the virus deposition solution also had an effect on virus inactivation when N95 respirators are stored under ambient conditions in health care settings. MS2 and phi6 suspended in DMEM-A, PBS, PBS with 0.1785% BSA, and saliva were deposited on N95 respirator coupons as described above for the elevated temperature experiments. Coupons were then incubated in the temperature- and humidity-controlled oven at 20°C and 36% RH for 24 h. Infective virus concentrations on coupons following 24 h of incubation were compared to the infective virus concentrations on control coupons that were determined immediately after droplets were dried for 1 h. Three independent replicates of the experiment were conducted.

### Virus extraction.

To recover viruses from control and treated coupons, the coupons were cut into 4 to 6 pieces with sterilized scissors and suspended in 1.3 ml elution medium. For phi6 and MS2, the elution medium consisted of 1.3 ml of 1% BSA (Dot Scientific, cat. no. DSA30075) in PBS. For MHV, the elution solution was DMEM-B. For IAV, the elution solution was IAV titer medium (same as DMEM-A, except 1% BSA is used in place of 0.1875% BSA). The coupon suspensions were vortexed at medium speed for 1 min. Viruses extracted in the elution buffer were enumerated as described above. To assess recovery from the coupons, 50 μl of the virus deposition solution was suspended into 1.3 ml of 1× PBS with 1% BSA, DMEM-B, or IAV titer medium for the phages, MHV, or IAV, respectively. Virus recovery was determined as the ratio of the control coupon virus titer to that of the suspended virus solution and were greater than 7% for all viruses and conditions (Text S1).

### Statistical analyses.

Unpaired *t* tests were performed to determine differences in virus inactivation for different treatment conditions and viruses using GraphPad Prism 8 software. Statistical significance was considered a *P* value of <0.05.
