# Supplementary material for: Humidity and Deposition Solution Play a Critical Role in Virus Inactivation by Heat Treatment of N95 Respirators
Source: mSphere. 2020 Oct 21;5(5):e00588-20. doi: 10.1128/mSphere.00588-20 (PMC7580954; doi:10.1128/mSphere.00588-20)
Supplement: TABLE S1 [file mSphere.00588-20-st001.pdf]

Virus	Relative humidity (%)	72°C[a]				82°C[a]			
		PBS	DMEM[b]	Saliva	PBS + BSA	PBS	DMEM	Saliva	PBS + BSA
MS2	1	0.11 0.36 (0.24)	1.31 1.57 (1.44)	-	-	0.08 0.29 (0.19)	2.42 3.12 (2.77)	0.73 1.02 (0.88)	0.90 0.64 (0.77)
	13	0.41 0.34 (0.38)	5.02 4.75 (4.89)	1.01 0.96 (0.99)	1.53 1.46 (1.5)	1.36 0.96 (1.16)	5.54 > 6.35 (5.93)	1.44 2.03 (1.74)	3.28 3.64 (3.46)
	25	1.31 1.46 (1.39)	6.41 6.28 (6.35)	1.41 1.48 (1.45)	2.25 3.18 (2.72)	4.07 4.03 (4.05)	> 6.86 > 6.18 (> 6.52)	-	-
	36	3.71 3.72 (3.72)	> 6.67 > 6.74 (> 6.71)	-	-	> 6.71 6.03 (> 6.37)	> 6.80 > 6.75 (> 6.78)	-	-
	48	> 6.81 6.64 (> 6.73)	> 6.57 > 6.19 (> 6.38)	-	-	> 6.78 > 6.32 (> 6.55)	> 6.58 > 5.48 (> 6.03)	-	-
	71	> 6.85 > 6.62 (> 6.74)	> 6.86 > 6.43 (> 6.65)	-	-	> 6.58 > 7.09 (> 6.84)	> 6.83 > 7.13 (> 6.98)	-	-
	89	> 7.11 > 6.62 (> 6.87)	> 6.93 > 6.19 (> 6.56)	-	-	> 6.83 > 6.96 (> 6.90)	>6.99 > 7.33 (> 7.16)	-	-
phi6	1	0.85 1.12 (0.99)	2.77 2.38 (2.58)	-	-	0.79 2.16 (1.48)	3.08 4.66 (3.87)	1.11 1.06 (1.09)	1.07 0.44 (0.76)
	13	1.97 1.27 (1.62)	4.48 4.12 (4.3)	1.19 0.70 (0.95)	1.26 1.40 (1.33)	3.84 2.97 (3.41)	6.61 7.18 (6.9)	2.33 2.91 (2.62)	2.44 1.51 (1.98)
	25	3.07 2.63 (2.85)	6.02 > 7.44 (> 6.73)	1.78 1.60 (1.69)	1.11 1.56 (1.34)	5.60 5.38 (5.49)	>8.08 > 7.18 (> 7.63)	-	-
	36	4.88 5.65 (5.27)	> 7.70 > 7.41 (> 7.56)	-	-	> 7.25 > 6.30 (> 6.78)	> 7.40 > 6.38 (> 6.89)	-	-
	48	7.14 7.03 (7.09)	> 7.76 > 7.45 (> 7.61)	-	-	> 6.97 >6.49 (> 6.73)	> 7.55 > 5.95 (> 6.75)	-	-
	71	> 6.20 > 7.05 (> 6.63)	> 7.83 > 7.10 (> 7.47)	-	-	> 7.08 > 7.02 (> 7.05)	> 7.40 > 7.54 (> 7.47)	-	-
	89	> 6.86 > 6.72 (> 6.79)	> 7.62 > 6.00 (> 6.81)	-	-	> 7.07 > 6.33 (> 6.70)	> 7.74 > 7.51 (> 7.63)	-	-
IAV	1	-	2.56 2.45 (2.51)	-	-	-	3.85 2.75 (3.30)	-	-
	13	-	3.98 3.50 (3.74)	-	-	-	> 3.96 4.14 (> 4.05)	-	-
	25	-	3.61 > 4.05 (> 3.83)	-	-	-	> 3.89 > 4.49 (> 4.19)	-	-
	89	-	> 4.19 > 4.19 (> 4.19)	-	-	-	> 4.33 > 4.43 (> 4.38)	-	-
MHV	1	-	0.89 1.60 (1.25)	-	-	-	2.89 2.53 (2.71)	-	-
	13	-	2.56 2.48 (2.52)	-	-	-	> 4.17 > 3.45 (> 3.81)	-	-
	25	-	> 3.52 > 3.59 (> 3.56)	-	-	-	> 3.56 > 3.31 (> 3.44)	-	-
	89	-	> 3.83 > 3.59 (> 3.71)	-	-	-	> 3.30 > 3.43 (> 3.37)	-	-

[a]Replicates shown side-by-side for a given treatment, and arithmetic mean shown in parentheses below replicates. Greater than symbol is used to designate replicates outside of dynamic range. For treatment settings with one or more replicates outside of dynamic range, the arithmetic mean was calculated from the values shown.

[b]DMEM-A was used as the deposition solution for MS2, phi6, and IAV experiments, and DMEM-B was used as the deposition solution for MHV experiments.
