# Supplementary material for: Humidity and Deposition Solution Play a Critical Role in Virus Inactivation by Heat Treatment of N95 Respirators
Source: mSphere. 2020 Oct 21;5(5):e00588-20. doi: 10.1128/mSphere.00588-20 (PMC7580954; doi:10.1128/mSphere.00588-20)
Supplement: TABLE S2 [file mSphere.00588-20-st002.pdf]

Virus	Treatment		72°C versus 82°C
	Deposition Solution	RH (%)	
MS2	PBS	1	0.306 (0.79)
		13	3.87 (0.061)
		25	34.3 **(0.0008)**
	DMEM-A	1	3.56 (0.071)
	Saliva	13	2.53 (0.13)
	PBS + BSA	13	10.7 **(0.0086)**
phi6	PBS	1	0.702 (0.56)
		13	3.20 (0.086)
		25	10.73 **(0.0086)**
	DMEM-A	1	1.59 (0.25)
		13	7.79 **(0.017)**
	Saliva	13	4.41 **(0.048)**
	PBS + BSA	13	1.37 (0.30)
IAV	DMEM-A	1	1.44 (0.29)
MHV	DMEM-B	1	3.68 (0.067)

[a]Significant p-values are indicated in bold (significance considered $p < 0.05$).
