# Supplementary material for: Humidity and Deposition Solution Play a Critical Role in Virus Inactivation by Heat Treatment of N95 Respirators
Source: mSphere. 2020 Oct 21;5(5):e00588-20. doi: 10.1128/mSphere.00588-20 (PMC7580954; doi:10.1128/mSphere.00588-20)
Supplement: TABLE S3 [file mSphere.00588-20-st003.pdf]

Temperature (°C)	Virus	Matrix	t-test (p-value)[a]			
			1% versus 13% RH	13% versus 25% RH	25% versus 36% RH	36% versus 48% RH
72	MS2	PBS	1.08 (0.39)	12.2 **(0.0066)**	31.0 **(0.001)**	NA
		DMEM-A	18.38 **(0.0029)**	9.74 **(0.010)**	NA	NA
	phi6	PBS	1.69 (0.23)	2.98 (0.10)	5.45 **(0.032)**	4.68 **(0.043)**
		DMEM-A	6.50 **(0.023)**	NA	NA	NA
	IAV	DMEM-A	5.02 **(0.038)**	NA	-	-
	MHV	DMEM-B	3.57 (0.070)	NA	-	-
82	MS2	PBS	4.32 (0.050)	14.4 **(0.0048)**	NA	NA
		DMEM-A	5.93 **(0.027)**	NA	NA	NA
	phi6	PBS	2.38 (0.14)	4.65 **(0.043)**	NA	NA
		DMEM-A	3.60 (0.069)	NA	NA	NA

[a]Significant p-values are indicated in bold (significance considered $p < 0.05$).

NA = Not assessed, because values were below detection limits.
