# Supplementary material for: Humidity and Deposition Solution Play a Critical Role in Virus Inactivation by Heat Treatment of N95 Respirators
Source: mSphere. 2020 Oct 21;5(5):e00588-20. doi: 10.1128/mSphere.00588-20 (PMC7580954; doi:10.1128/mSphere.00588-20)
Supplement: TABLE S4 [file mSphere.00588-20-st004.pdf]

Treatment			t-test (p-value)[a]					
Deposition solution	Temperature (°C)	RH (%)	MS2 versus phi6	MS2 versus IAV	MS2 versus MHV	phi6 versus IAV	phi6 versus MHV	IAV versus MHV
DMEM	72	1	4.84 **(0.040)**	7.55 **(0.017)**	0.52 (0.66)	0.346 (0.76)	3.28 (0.082)	3.51 (0.073)
		13	2.60 (0.12)	4.16 (0.053)	16.8 **(0.0035)**	1.87 (0.20)	9.65 **(0.011)**	5.01 **(0.038)**
	82	1	1.27 (0.33)	0.813 (0.50)	0.152 (0.89)	0.592 (0.61)	1.43 (0.29)	1.02 (0.42)
PBS	72	1	4.08 (0.055)	-	-	-	-	-
		13	3.54 (0.071)	-	-	-	-	-
		25	6.30 **(0.024)**	-	-	-	-	-
		36	4.03 (0.057)	-	-	-	-	-
	82	1	4.68 **(0.043)**	-	-	-	-	-
		13	1.14 (0.37)	-	-	-	-	-
		25	5.43 **(0.032)**	-	-	-	-	-
Saliva	72	13	0.162 (0.89)	-	-	-	-	-
		25	2.54 (0.13)	-	-	-	-	-
	82	1	1.43 (0.29)	-	-	-	-	-
		13	2.14 (0.17)	-	-	-	-	-
PBS + BSA	72	13	2.1 (0.17)	-	-	-	-	-
		25	2.7 (0.12)	-	-	-	-	-
	82	1	0.0440 (0.97)	-	-	-	-	-
		13	2.98 (0.10)	-	-	-	-	-

[a]Significant p-values are indicated in bold (significance considered $p < 0.05$).
