# Supplementary figures and images for: Humidity and Deposition Solution Play a Critical Role in Virus Inactivation by Heat Treatment of N95 Respirators
Source: mSphere. 2020 Oct 21;5(5):e00588-20. doi: 10.1128/mSphere.00588-20 (PMC7580954; doi:10.1128/mSphere.00588-20)

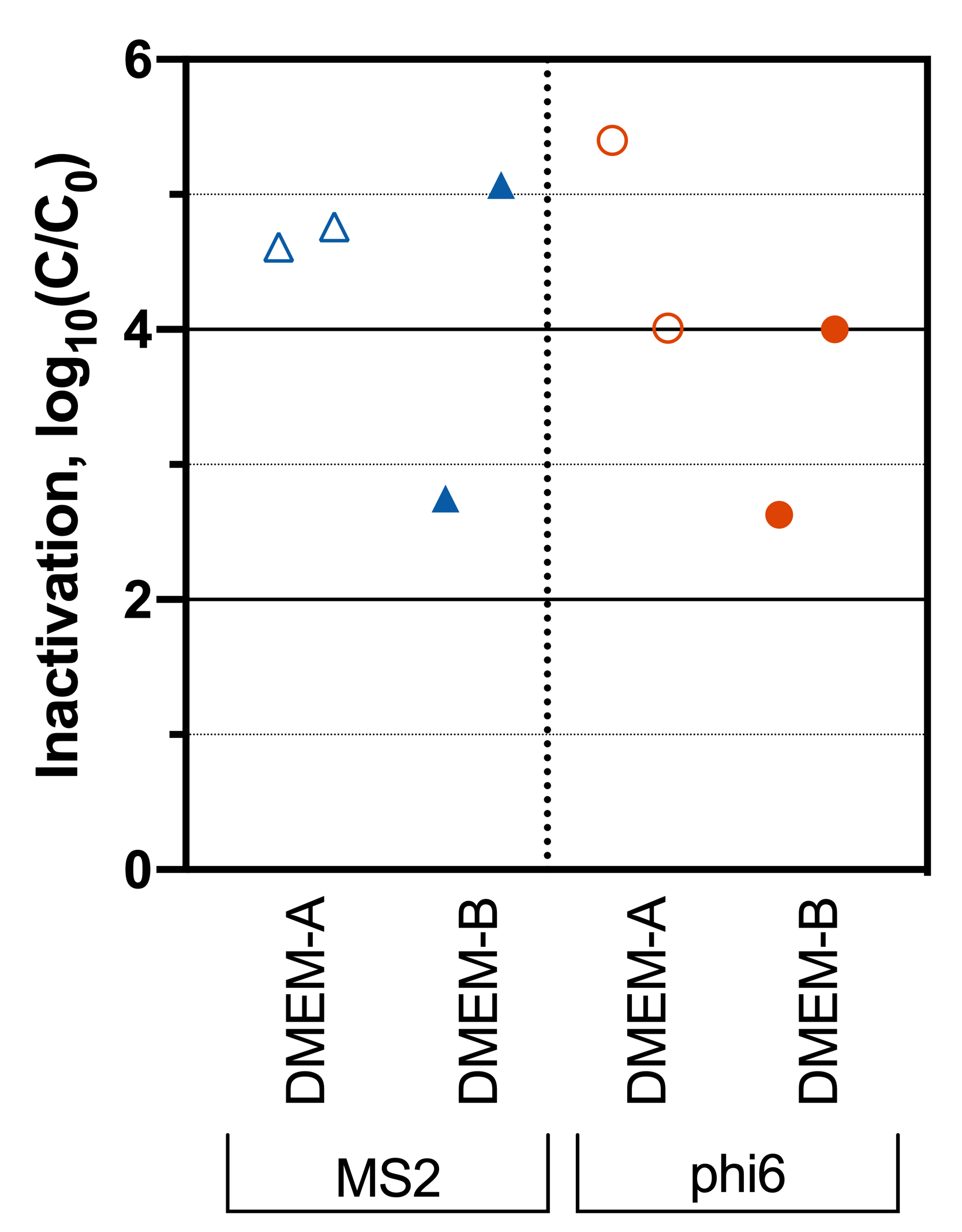

Supplement: FIG S1 [file mSphere.00588-20-sf001.tif]

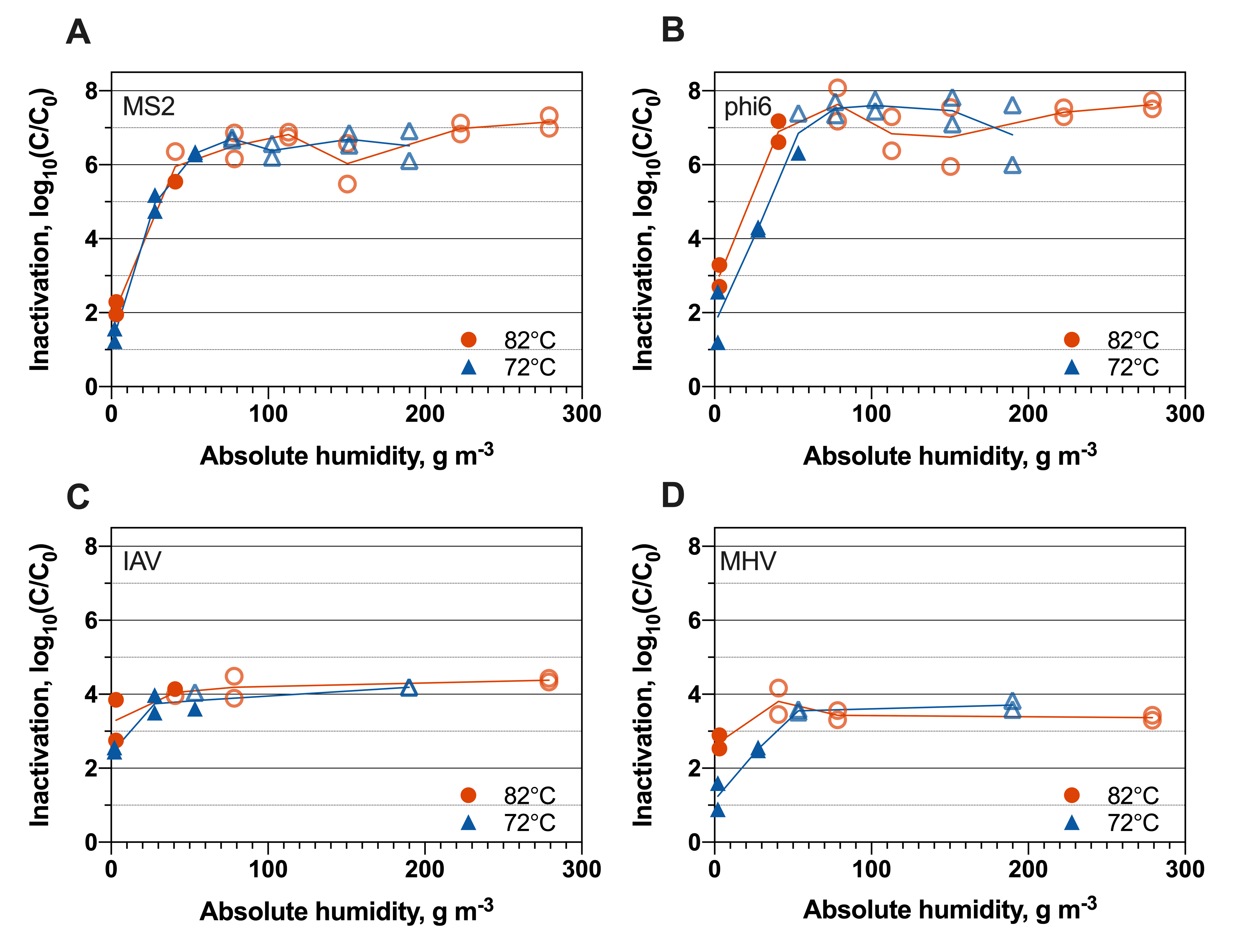

Supplement: FIG S2 [file mSphere.00588-20-sf002.tif]
