# Supplementary material for: Humidity and Deposition Solution Play a Critical Role in Virus Inactivation by Heat Treatment of N95 Respirators
Source: mSphere. 2020 Oct 21;5(5):e00588-20. doi: 10.1128/mSphere.00588-20 (PMC7580954; doi:10.1128/mSphere.00588-20)
Supplement: TABLE S5 [file mSphere.00588-20-st005.pdf]

Component	Type	DMEM-A	DMEM-B
DMEM			
Glycine	Amino Acid	0.4 mM	0.4 mM
L-Arginine hydrochloride	Amino Acid	0.4 mM	0.4 mM
L-Cystine 2HCl	Amino Acid	0.2 mM	0.2 mM
L-Glutamine	Amino Acid	4 mM	2 mM*
L-Histidine hydrochloride-H_2O	Amino Acid	0.2 mM	0.2 mM
L-Isoleucine	Amino Acid	0.8 mM	0.8 mM
L-Leucine	Amino Acid	0.8 mM	0.8 mM
L-Lysine hydrochloride	Amino Acid	0.8 mM	0.8 mM
L-Methionine	Amino Acid	0.2 mM	0.2 mM
L-Phenylalanine	Amino Acid	0.4 mM	0.4 mM
L-Serine	Amino Acid	0.4 mM	0.4 mM
L-Threonine	Amino Acid	0.8 mM	0.8 mM
L-Tryptophan	Amino Acid	0.08 mM	0.08 mM
L-Tyrosine disodium salt dihydrate	Amino Acid	0.4 mM	0.4 mM
L-Valine	Amino Acid	0.8 mM	0.8 mM
Choline chloride	Vitamin	0.03 mM	0.03 mM
D-Calcium pantothenate	Vitamin	0.01 mM	0.008 mM
Folic acid	Vitamin	0.01 mM	0.009 mM
i-Inositol	Vitamin	0.04 mM	0.04 mM
Niacinamide	Vitamin	0.03 mM	0.03 mM
Pyridoxine hydrochloride	Vitamin	0.02 mM	0.02 mM
Riboflavin	Vitamin	0.001 mM	0.001 mM
Thiamine hydrochloride	Vitamin	0.01 mM	0.01 mM
Calcium Chloride ($CaCl_2$) (anhyd.)	Inorganic Salt	1.8 mM	1.8 mM
Ferric Nitrate ($Fe(NO_3)_3$-$9H_2O$)	Inorganic Salt	0.0002 mM	0.0002 mM
Magnesium Sulfate ($MgSO_4$) (anhyd.)	Inorganic Salt	0.8 mM	0.8 mM
Potassium Chloride (KCl)	Inorganic Salt	5.3 mM	5.4 mM
Sodium Bicarbonate ($NaHCO_3$)	Inorganic Salt	44 mM	44 mM
Sodium Chloride (NaCl)	Inorganic Salt	110.3 mM	109.5 mM
Sodium Phosphate monobasic (NaH_2PO_4-H_2O)	Inorganic Salt	0.9 mM	0.9 mM
D-Glucose (Dextrose)	Other	25 mM	25 mM
Phenol red	Other	0.04 mM	0.04 mM
HEPES (N-2-hydroxyethylpiperazine-N-2-ethane sulfonic acid)	Organic Chemical	25 mM	-
Bovine serum albumin (Fraction V)	Protein	0.19%	-
Horse serum	Protein	-	2%
Penicillin/Streptomycin	Antibiotics	1%	1%

**Not in DMEM, added separately.*
