# Supplementary material for: Humidity and Deposition Solution Play a Critical Role in Virus Inactivation by Heat Treatment of N95 Respirators
Source: mSphere. 2020 Oct 21;5(5):e00588-20. doi: 10.1128/mSphere.00588-20 (PMC7580954; doi:10.1128/mSphere.00588-20)
Supplement: TABLE S6 [file mSphere.00588-20-st006.pdf]

Temperature (°C)	Virus	RH (%)	t-test (p-value)[a]				
			PBS versus saliva	PBS versus PBS+BSA	PBS versus DMEM-A	DMEM-A versus saliva	DMEM-A versus PBS+BSA
72	MS2	**1**	-	-	6.68 **(0.022)**	-	-
		**13**	14.2 **(0.0049)**	22.6 **(0.0019)**	32.3 **(0.0010)**	28.4 **(0.0012)**	24.3 **(0.0017)**
		**25**	0.725 (0.54)	2.82 (0.11)	50.0 **(0.0004)**	66.40 **(0.0002)**	7.73 **(0.016)**
	phi6	**1**	-	-	6.70 **(0.022)**	-	-
		**13**	1.58 (0.25)	0.81 (0.50)	6.81 **(0.021)**	11.0 **(0.0081)**	15.4 **(0.0042)**
		**25**	4.88 **(0.040)**	4.81 **(0.041)**	NA	NA	NA
82	MS2	**1**	3.85 (0.061)	3.50 (0.073)	7.07 **(0.019)**	5.00 **(0.038)**	5.36 **(0.033)**
		**13**	1.61 (0.25)	8.55 **(0.013)**	NA	NA	NA
	phi6	**1**	0.569 (0.63)	0.955 (0.44)	2.29 (0.15)	3.52 (0.072)	3.66 (0.067)
		**13**	1.50 (0.27)	2.25 (0.15)	6.71 **(0.021)**	10.5 **(0.0089)**	9.02 **(0.012)**

[a]Significant p-values are indicated in bold (significance considered $p < 0.05$).

NA = Not assessed, because values were below detection limits.
