# Supplementary material for: Humidity and Deposition Solution Play a Critical Role in Virus Inactivation by Heat Treatment of N95 Respirators
Source: mSphere. 2020 Oct 21;5(5):e00588-20. doi: 10.1128/mSphere.00588-20 (PMC7580954; doi:10.1128/mSphere.00588-20)
Supplement: TABLE S7 [file mSphere.00588-20-st007.pdf]

Matrix	Description
DMEM-A	DMEM supplemented with 0.1875% Fraction V BSA, 25 mM HEPES, and 1% antibiotics (Table S2)
DMEM-B	DMEM supplemented with 2% horse serum, L-glutamine, and 1% antibiotics (Table S2)
PBS	1X phosphate buffered saline (Invitrogen)
PBS + BSA	1X phosphate buffered saline (Invitrogen) supplemented with 0.1875% Fraction V BSA
Saliva	UV_{254} sterilized fresh saliva collected from human volunteers
