# Supplementary material for: Humidity and Deposition Solution Play a Critical Role in Virus Inactivation by Heat Treatment of N95 Respirators
Source: mSphere. 2020 Oct 21;5(5):e00588-20. doi: 10.1128/mSphere.00588-20 (PMC7580954; doi:10.1128/mSphere.00588-20)
Supplement: TEXT S1 [file mSphere.00588-20-s0001.pdf]

Supplemental Text S1.

Control experiment to test the potential impact of drying time. The MS2 and phi6 virus stock was deposited on coupons and allowed to dry for either one or three hours prior to the heat treatment. The results from this control experiment confirmed that the $\log_{10}$ inactivation observed through heat treatment was not affected by the drying time of virus droplets on the coupon (Figure S1).

Virus recovery from N95 respirator coupons. To assess virus recovery from N95 respirator coupons, 50 uL of each of the virus stocks was suspended into their respective elution solutions. The virus suspensions were extracted and enumerated with the same methods used on the coupons. Recovery rates were calculated as the ratio of the control coupon titer to the virus suspension. Average recovery rates for each virus tested are 56% for IAV, 28% for MHV, 38% for MS2 suspended in PBS, 34% for MS2 suspended in DMEM-A, 104% for phi6 suspended in PBS, and 7% for phi6 suspended in DMEM-A.
